# Effectiveness and safety of pegylated liposomal doxorubicin versus epirubicin as neoadjuvant or adjuvant chemotherapy for breast cancer: a real-world study

**DOI:** 10.1186/s12885-021-09050-6

**Published:** 2021-12-06

**Authors:** Jin Zhang, Hongchuan Jiang, Jian Zhang, Guoqiang Bao, Guoqiang Zhang, Haibo Wang, Xi Wang

**Affiliations:** 1 Third Department of Breast Surgery, Tianjin Medical University Cancer Institute and Hospital; National Clinical Research Center for Cancer; Key Laboratory of Cancer Prevention and Therapy; Clinical Research Center for Cancer, Tianjin, 300060 China; 2 Department of Breast Surgery, Beijing Chaoyang Hospital, Capital Medical University, Beijing, 100020 China; 3 Department of Medical Oncology, Fudan University Shanghai Cancer Center, Shanghai, 200032 China; 4 General Surgery Department, Tangdu Hospital, The Fourth Military Medical University, Xi’an, 710038 China; 5 Department of Breast Surgery, Harbin Medical University Cancer Hospital, Harbin, 150040 China; 6 Department of Breast Surgery, The Affiliated Hospital of Qingdao University, Qingdao, 266071 China; 7 Department of Breast Surgery, Sun Yat-sen University Cancer Center, Guangzhou, 510060 China

**Keywords:** Adjuvant chemotherapy, Breast cancer, Epirubicin, Neoadjuvant chemotherapy, Pegylated liposomal doxorubicin

## Abstract

**Background:**

Pegylated liposomal doxorubicin (PLD) is an improved formulation of doxorubicin with comparable effectiveness but significantly lower cardiotoxicity than conventional anthracycline. This study aimed to evaluate the real-world effectiveness and safety of PLD versus epirubicin as neoadjuvant or adjuvant treatment for breast cancer.

**Methods:**

Clinical data of invasive breast cancer patients who received neoadjuvant or adjuvant chemotherapy with PLD or epirubicin were retrospectively collected. Propensity score matching (PSM) was performed to reduce the risk of selection bias. The molecular typing of these patients included Luminal A, Luminal B, HER2-positive, and basal-like/triple-negative. The primary outcome was pathological complete response (pCR) rate for neoadjuvant chemotherapy and 3-year disease-free survival (DFS) rate for adjuvant chemotherapy. Noninferiority was suggested if the lower limit of the 95% CI for the 3-year DFS rate difference was greater than − 10%. The secondary outcome was adverse reactions.

**Results:**

A total of 1213 patients were included (neoadjuvant, *n* = 274; adjuvant, *n* = 939). pCR (ypT0/Tis ypN0) rates of patients who received neoadjuvant chemotherapy were 11.6% for the PLD group and 7.0% for the epirubicin group, but the difference was not statistically significant (*P* = 0.4578). The 3-year DFS rate of patients who received adjuvant chemotherapy was 94.9% [95%CI, 91.1–98.6%] for the PLD group and 95.4% [95%CI, 93.0–97.9%] for the epirubicin group (*P* = 0.5684). Rate difference between the two groups and its 95% CI was - 0.55 [− 5.02, 3.92]. The lower limit of the 95% CI was − 5.0% > − 10.0%, suggesting that PLD is not be inferior to epirubicin in adjuvant chemotherapy for breast cancer. The incidences of myelosuppression, decreased appetite, alopecia, gastrointestinal reactions, and cardiotoxicity were lower in the PLD group than in the epirubicin group, while the incidence of nausea was higher in the PLD group.

**Conclusions:**

In the neoadjuvant and adjuvant treatment of breast cancer, effectiveness is similar but toxicities are different between the PLD-containing regimen and epirubicin-containing regimen. Therefore, further study is warranted to explore PLD-based neoadjuvant and adjuvant chemotherapy for breast cancer.

## Background

Breast cancer is the most common malignancy in women worldwide. According to the 2018 statistics of the International Agency for Research on Cancer (IARC) of the World Health Organization, there were 2.08 million new cases of breast cancer and 620,000 breast cancer-related deaths in the world, which accounted for 24.2 and 15% of all malignancies and malignancy-related deaths in women, respectively [1]. Preoperative neoadjuvant chemotherapy and postoperative adjuvant chemotherapy can effectively reduce the risk of recurrence and improve the cure rate of early and locally advanced breast cancer patients [2, 3].

Anthracycline-based chemotherapy is a common neoadjuvant and adjuvant therapy for breast cancer patients. The recommended anthracycline drugs include doxorubicin and epirubicin [4]. Anthracyclines have significant effectiveness in breast cancer, but they often cause alopecia, myelosuppression, and gastrointestinal reactions. In addition, anthracycline-induced cardiotoxicity was reported to be closely associated with the cumulative dose of the drug [5], and can also occur at a low dose, and can be acute, chronic, and delayed, most of which occur in the first year of treatment [6]. The risk factors for anthracycline-induced cardiotoxicity include being < 5 or > 65 years of age, past or current chest irradiation, history of heart diseases, or the presence of cardiovascular risk factors [7]. Furthermore, concurrent anti-HER2 therapies can increase the risk of cardiotoxicity with anthracyclines [8–10]. Anthracycline-related cardiotoxicities are often progressive and irreversible, leading to ventricular dysfunction, heart failure, and arrhythmia [11].

Pegylated liposomal doxorubicin (PLD) is a liposomal formulation of doxorubicin with comparable effectiveness but markedly lower cardiotoxicity than conventional anthracycline [12], thus allowing a higher cumulative dose of the drug. The National Comprehensive Cancer Network guidelines recommended PLD as the first-line treatment for advanced breast cancer [4]. A phase II clinical trial compared the effectiveness of PLD versus epirubicin in combination with vinorelbine as the first-line treatment for metastatic breast cancer. The study found that there were no significant differences in ORR, PFS, and OS between the two groups. Furthermore, while cardiotoxicity was not reported in the PLD group, one (1.9%) patient reported arrhythmia and two (3.7%) patients had over 20% decrease in LVEF in the epirubicin group [13]. Several research groups have explored the effectiveness and safety of PLD as neoadjuvant/adjuvant therapy for breast cancer. Song et al. carried out a phase I/II trial of PLD neoadjuvant therapy for breast cancer. The results showed that the maximum tolerated dose of PLD was 40 mg/m^2^, and the breast pCR rate was 18.8% (95% CI, 11.5–26.0%) with no significant decrease in LVEF [14, 15]. Another multicenter randomized-controlled trial confirmed that PLD and trastuzumab combination therapy significantly lowered the incidence of cardiotoxicity compared with doxorubicin plus cyclophosphamide followed by paclitaxel plus trastuzumab [16].

This real-world study aimed to compare the effectiveness and safety of PLD to epirubicin as neoadjuvant or adjuvant treatment for breast cancer patients.

## Methods

### Data source and study population

The medical records of breast cancer patients who received PLD (CSPC Ouyi Pharmaceutical Co., Ltd., Shijiazhuang, China) or epirubicin-based neoadjuvant (January 2014 to January 2018) or adjuvant treatment (June 2014 to June 2016) were retrospectively collected. Inclusion criteria: 18–70 years old; female; histologically confirmed invasive breast cancer; received PLD or epirubicin neoadjuvant chemotherapy or adjuvant chemotherapy; LVEF ≥50%. Exclusion criteria: occult breast cancer patients; used two or more anthracyclines during neoadjuvant or adjuvant chemotherapy; previously received other chemotherapy regimens. After screening, the patients were divided into the neoadjuvant chemotherapy group and adjuvant chemotherapy group according to their treatment stage and then into the PLD group and epirubicin group according to the drug regimen.

The study was approved by the Ethics Research Committee of Tianjin Cancer Hospital.

Trial Registration: ClinicalTrials.gov, Identifier: NCT03983096.

### Molecular subtyping

The expression statuses of ER, PR, HER2, and Ki67 were detected by immunohistochemical staining to determine the molecular subtyping. ER and PR were considered positive when more than 1% of the tumor cells exhibited positive staining. For HER2 staining, a score of 3+ was considered positive; a specimen with a score of 2+ was tested by fluorescence in situ hybridization analysis. The standard threshold value of Ki67 was 20%. If ≥20%, it was considered to be high Ki67 expression, otherwise low Ki67 expression. In 2013, the St. Gallen International Breast Cancer Conference defined the molecular classification of breast cancer [17]. When ER and/or PR+, HER2- and Ki67 < 20%, it was defined as Luminal A. Luminal B was divided into two situations. When ER+ and/or PR < 20%, HER2- and Ki67 ≥ 20%, it was defined as HER2-negative (B1 type). When ER+ and/or PR+, HER2 overexpression and Ki67 ≥ 20%, it was defined as HER2-positive type (B2 type). The characteristics of HER2-positive type were HER2+, ER- and PR-. Basal-like/triple-negative features were HER2-, ER- and PR-.

### Cardiotoxicity

Cardiotoxicity was defined as abnormal results of cardiac function in clinical evaluation, including decreased ventricular ejection fraction (LVEF ≤50%; LVEF lower than ≥10% of the baseline value), congestive heart failure, arrhythmia, etc. [7]. Cardiotoxicity was not graded.

### Statistical analysis

The primary outcome for neoadjuvant chemotherapy was the total pathological complete response (pCR) rate (tpCR, ypT0/Tis ypN0), which was defined as the absence of residual invasive cancer cells or only carcinoma in situ in the primary and metastatic lymph nodes after surgery. The primary outcome for adjuvant chemotherapy was the 3-year disease-free survival (DFS) rate. DFS referred to the time from the first postoperative chemotherapy to recurrence, metastasis, or death. The secondary outcome was the incidence of adverse reactions.

When the baseline characteristics of the eligible patients were balanced, the eligible case data were used for statistical analysis. Otherwise, propensity score-matching (PSM) was conducted to reduce the selection bias between the PLD and epirubicin groups. The variables included in PSM were age, lymph node metastasis, tumor size, and molecular typing.

All statistical tests were two-sided with a significance level of α = 0.05. The 3-year DFS rate of adjuvant chemotherapy was compared between the two groups using the chi-square test or Fisher’s exact test. The 95% confidence interval (CI) for the 3-year DFS rate difference was calculated. For exploratory purposes, a noninferiority test was performed, with the noninferiority margin set at − 10%. Noninferiority was suggested if the lower limit of the 95% CI for the 3-year DFS rate difference was greater than − 10% (based on clinical considerations). The pCR rate of neoadjuvant chemotherapy was also compared between the two groups using the chi-square test or Fisher’s exact test. According to the conditions of the patients included, this study conducted subgroup analyses of the main study outcomes for the neoadjuvant chemotherapy and adjuvant chemotherapy population. Factors such as menopausal status (premenopausal or postmenopausal), tumor size (T1 or T2), lymph node metastasis (N0, N1, N2, and N3), clinical stage (II or IIIA), ER status (positive or negative), PR status (positive or negative), HER2 status (positive or negative), and Ki-67 expression level (< 20% or ≥ 20%) were considered for the subgroup analyses.

For safety analysis, the number and incidence of adverse reactions in the PLD group and epirubicin group were counted.

## Results

### Patients

**Fig. 1:**
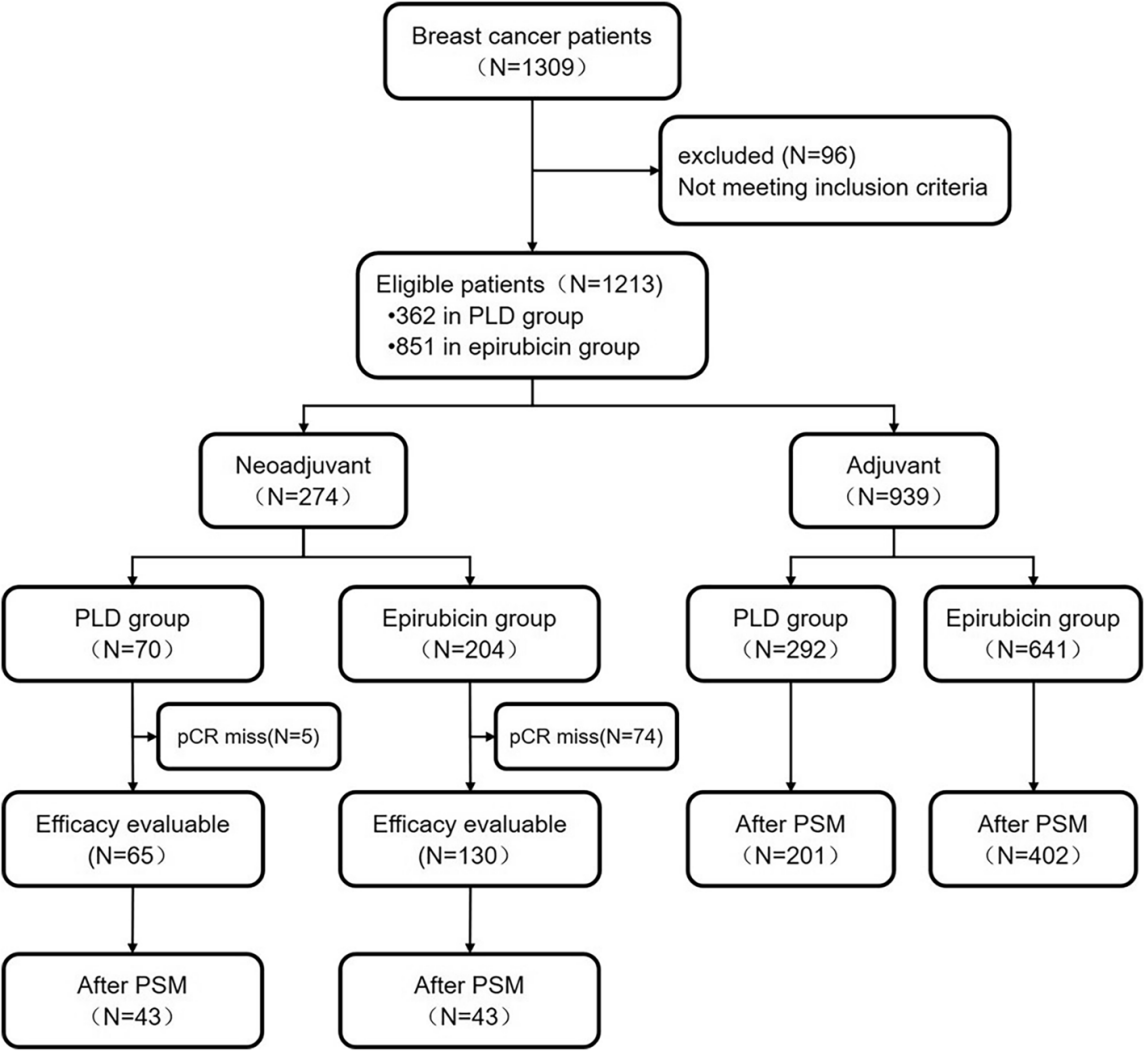
Flowchart showing patient selection. PLD, pegylated liposomal doxorubicin; pCR, pathological complete response; PSM, propensity score matching

The clinical data of 1309 breast cancer patients who were diagnosed and treated in seven hospitals in China between January 2014 and January 2018 were retrospectively reviewed. A total of 1213 patients met the selection criteria (patient selection flowchart shown in Fig. 1), including 274 neoadjuvant chemotherapy patients and 939 adjuvant chemotherapy patients.

The common chemotherapy regimens included PLD or epirubicin combined with cyclophosphamide (C), PLD or epirubicin combined with C followed by a taxane (T), T followed by PLD or epirubicin combined with C, PLD or epirubicin combined with T, PLD or epirubicin combined with T and C, and PLD or epirubicin combined with C and 5-fluorouracil (Table 1). The dose of treatment was 30–40 mg/m^2^ for PLD and 60–75 mg/m^2^ for epirubicin.

**Table 1:** Chemotherapy regimen

Chemotherapy regimen	PLD group	Epirubicin group
Neoadjuvant chemotherapy	70	204
PLD/Epirubicin+C, n (%)	7 (10.0%)	23 (11.3%)
PLD/Epirubicin+C-T, n (%)	28 (40.0%)	33 (16.2%)
PLD/Epirubicin+T, n (%)	14 (20.0%)	53 (26.0%)
T + PLD/Epirubicin+C, n (%)	25 (38.5%)	62 (30.4%)
others, n (%)	9 (12.9%)	33 (16.2%)
Adjuvant chemotherapy	292	647
PLD/Epirubicin+C, n (%)	88 (30.1%)	66 (10.2%)
PLD/Epirubicin+C + T, n (%)	22 (7.5%)	68 (10.5%)
PLD/Epirubicin+C-T, n (%)	136 (46.6%)	255 (39.4%)
C + PLD/Epirubicin+F, n (%)	1 (0.3%)	0 (0.0%)
T + PLD/Epirubicin, n (%)	20 (6.9%)	198 (30.6%)
T-PLD/Epirubicin+C, n (%)	8 (2.7%)	25 (3.9%)

PLD, pegylated liposomal doxorubicin; C, Cyclophosphamide; T, Taxane; F, 5-fluorouracil

Among the 274 patients who received neoadjuvant chemotherapy, 195 (71.2%) had evaluable effectiveness (79 patients discontinued neoadjuvant treatment due to unknown reasons and lacked the results of pCR), including 65 (65/195, 33.3%) patients who received PLD-containing regimen, and 130 (130/195, 66.7%) patients who received epirubicin-containing regimen. The baseline characteristics of the patients, including age < 35 (*P* = 0.0353), tumor size (*P* = 0.0452), and lymph node metastasis (*P* = 0.0109), were not evenly distributed across the PLD and epirubicin groups before PSM. After one-to-one PSM, there were 43 patients in each group, and the baseline characteristics of the two groups were balanced (Table 2). The median age was 49 (25–70) and 48 (27–67) years, the number of premenopausal patients was 20 (52.6%) and 27 (64.3%), and the number of patients with Ki67 ≥ 20% were 22 (75.9%) and 18 (72.0%) for the PLD group and epirubicin group, respectively.

**Table 2:** Baseline characteristics of neoadjuvant chemotherapy patients before and after PSM

Characteristics	Before PSM (*N* = 185)			After PSM (*N* = 86)		
	PLD group (*N* = 65)	Epirubicin group (*N* = 130)	*P* value	PLD group (*N* = 43)	Epirubicin group (*N* = 43)	*P* value
Age (year), n (%)			0.0353			1.0000
< 35	15 (23.1%)	15 (11.5%)		10 (23.3%)	10 (23.3%)	
≥ 35	50 (76.9%)	115 (88.5%)		33 (76.7%)	33 (76.7%)	
Age (year), Median (range)	46 (25,70)	50 (27,67)	0.1919	49 (36–60)	48 (36–58)	0.4812
Menopausal status, n (%)			0.1082			0.2903
Premenopausal	38 (58.5%)	64 (49.2%)		20 (46.5%)	27 (62.8%)	
Postmenopausal	22 (33.9%)	62 (47.7%)		18 (41.9%)	15 (34.9%)	
Missing	5 (7.7%)	4 (3.1%)		5 (11.6%)	1 (2.3%)	
Nodal status, n (%)			0.0109			0.8122
N0	22 (33.9%)	40 (30.8%)		16 (37.2%)	12 (27.9%)	
N1	21 (32.3%)	25 (19.2%)		14 (32.6%)	16 (37.2%)	
N2	7 (10.8%)	30 (23.1%)		7 (16.3%)	9 (20.9%)	
N3	7 (10.8%)	33 (25.4%)		6 (14.0%)	6 (14.0%)	
Missing	8 (12.3%)	2 (1.5%)				
Tumor size, n (%)			0.0452			0.9719
T1	14 (21.5%)	43 (33.1%)		14 (32.6%)	12 (27.9%)	
T2	30 (46.2%)	42 (32.3%)		24 (55.8%)	26 (60.5%)	
T3	4 (6.2%)	15 (11.5%)		3 (7.0%)	3 (7.0%)	
T4	2 (3.1%)	13 (10.0%)		2 (4.7%)	2 (4.7%)	
Missing	15 (23.1%)	17 (13.1%)				
Molecular subtype, n (%)			0.5592			0.5642
Luminal A	10 (15.4%)	18 (13.9%)		8 (18.6%)	5 (11.6%)	
Luminal B	30 (46.2%)	56 (43.1%)		23 (53.5%)	20 (46.5%)	
HER2+	6 (9.2%)	13 (10.0%)		4 (9.3%)	2(4.7%)	
BASAL-LIKE	3 (4.6%)	14 (10.8%)		1 (2.3%)	3 (7.0%)	
Missing	16 (24.6%)	29 (22.3%)		7 (16.3%)	13 (30.2%)	
Ki-67 expression, n (%)			0.5803			0.7468
< 20%	8 (12.3%)	16 (12.3%)		7 (16.3%)	7 (16.3%)	
≥ 20%	34 (52.3%)	52 (40.0%)		22 (51.2%)	18 (41.9%)	
Missing	23 (35.4%)	62 (47.7%)		14 (32.6%)	18 (41.9%)	

*PLD* Pegylated liposomal doxorubicin

Among the patients who received adjuvant chemotherapy, 292 (31.1%) patients received a PLD-containing regimen, and 647 (68.9%) patients received an epirubicin-containing regimen. The baseline characteristics of the patients, namely age < 35 (*P* = 0.0262) and lymph node metastasis (*P* = 0.0046), were not evenly distributed between the two groups before PSM. After PSM (1:2), there were 201 patients in the PLD group and 402 patients in the epirubicin group, and the baseline characteristics were balanced between the two groups (Table 3). The median age was 49 (25–69) years, and 50 (23–70) years, the number of premenopausal patients was 105 (56.8%) and 228 (58.6%), and the number of patients with Ki67 ≥ 20% was 125 (77.2%) and 242 (72.2%), respectively.

**Table 3:** Baseline characteristics of adjuvant chemotherapy patients before and after PSM

Characteristics	Before PSM (*N* = 939)			After PSM (*N* = 603)		
	PLD group (*N* = 292)	Epirubicin group (*N* = 647)	*P* value	PLD group (*N* = 201)	Epirubicin group (*N* = 402)	*P* value
Age (year), n (%)			0.0262			1.0000
< 35	14 (4.8%)	58 (9.0%)		10 (5.0%)	20 (5.0%)	
≥ 35	278 (95.2%)	589 (91.0%)		191 (95.0%)	382 (95.0%)	
Age (year), Median (range)	48 (25,70)	49 (23,70)	0.8529	49 (42–56)	50 (44–56)	0.7495
Menopausal status, n (%)			0.2343			0.6739
Premenopausal	115 (39.4%)	242 (37.4%)		105 (52.2%)	228 (56.7%)	
Postmenopausal	153 (52.4%)	384 (59.4%)		80 (39.8%)	161 (40.1%)	
Missing	24 (8.2%)	21 (3.3%)		16 (8.0%)	13 (3.2%)	
Histological grade, n (%)			0.9289			0.4268
I	3 (1.0%)	9 (1.4%)		2 (1.0%)	9 (2.2%)	
II	121 (41.4%)	294 (45.4%)		95 (47.3%)	206 (51.2%)	
III	77 (26.4%)	181 (28.0%)		52 (25.9%)	95 (23.6%)	
Missing	91 (31.2%)	163 (25.2%)		52 (25.9%)	92 (22.9%)	
Nodal status, n (%)			0.0046			0.9049
N0	141 (48.3%)	284 (43. 9%)		103 (51.2%)	202 (50.3%)	
N1	80 (27.4%)	184 (28.4%)		69 (34.3%)	202 (50.3%)	
N2	22 (7.5%)	89 (13.8%)		19 (9.5%)	36 (9.0%)	
N3	12 (4.1%)	58 (9.0%)		10 (5.0%)	26 (6.5%)	
Missing	37 (12.7%)	32 (5.0%)		0 (0.0%)	0 (0.0%)	
Tumor size, n (%)			0.3915			0.4213
T1	130 (44.5%)	268 (41.4%)		97 (48.3%)	182 (45.3%)	
T2	105 (36.0%)	277 (42.8%)		81 (40.3%)	179 (44.5%)	
T3	7 (2.4%)	16 (2.5%)		7 (3.5%)	8 (2.0%)	
T4	1 (0.3%)	5 (0.8%)		1 (0.5%)	1 (0.3%)	
Missing	49 (16.8%)	81 (12.5%)		15 (7.5%)	32 (8.0%)	
Molecular subtype, n (%)			0.2054			0.7747
Luminal A	56 (19.2%)	91 (14.1%)		40 (19.9%)	76 (18.9%)	
Luminal B	141 (48.3%)	317 (49.0%)		126 (62.7%)	261 (64.9%)	
HER2+	32 (11.0%)	76 (11.8%)		20 (10.0%)	43 (10.7%)	
BASAL-LIKE	17 (5.8%)	51 (7.9%)		15 (7.5%)	22 (5.5%)	
Missing	46 (15.8%)	112 (17.3%)		0 (0.0%)	0 (0.0%)	
Ki-67 expression, n (%)			0.3344			0.2419
< 20%	65 (22.3%)	122 (18.9%)		37 (18.4%)	93 (23.1%)	
≥ 20%	174 (59.6%)	388 (60.0%)		125 (62.2%)	242 (60.2%)	
Missing	53 (18.2%)	137 (21.2%)		39 (19.4%)	67 (16.7%)	

PLD, pegylated liposomal doxorubicin

### Effectiveness

#### Pathological complete response

Before PSM, the postoperative pathology of 195 evaluable patients who received neoadjuvant chemotherapy showed slightly higher tpCR in the PLD group (9, 13.9%) than in the epirubicin group (12, 9.2%), but the difference was not statistically significant (*P* = 0.3270). Breast pCR (bpCR, ypT0/Tis) was also higher in the PLD group (16, 24.6%) than in the epirubicin group (20, 15.4%), but the difference was not statistically significant (*P* = 0.1173) (Table 4).

**Table 4:** pCR of neoadjuvant chemotherapy

pCR	PLD group n (%)	Epirubicin group n (%)	*P* value
Before PSM, n	65	130	
bpCR	16 (24.6%)	20 (15.4%)	0.1173
tpCR	9 (13.9%)	12 (9.2%)	0.3270
After PSM, n	43	43	
bpCR	11 (25.6%)	6 (14.0%)	0.1758
tpCR	5 (11.6%)	3 (7.0%)	0.4578

*PLD* Pegylated liposomal doxorubicin, *tpCR* Total pathological complete response, *bpCR* Breast pathological complete response

After PSM, tpCR (11.6% vs. 7.0%, *P* = 0.4578) and bpCR (25.6% vs. 14.0%, *P* = 0.1758) were also comparable between the PLD group and epirubicin group (Table 4).

Given that there were fewer cases in the neoadjuvant chemotherapy group, the subgroup analyses (menopausal status, tumor size, N status, clinical stage, ER status, PR status, HER2 status, and Ki-67 expression level) could not be performed.

#### Three-year DFS

**Fig. 2:**
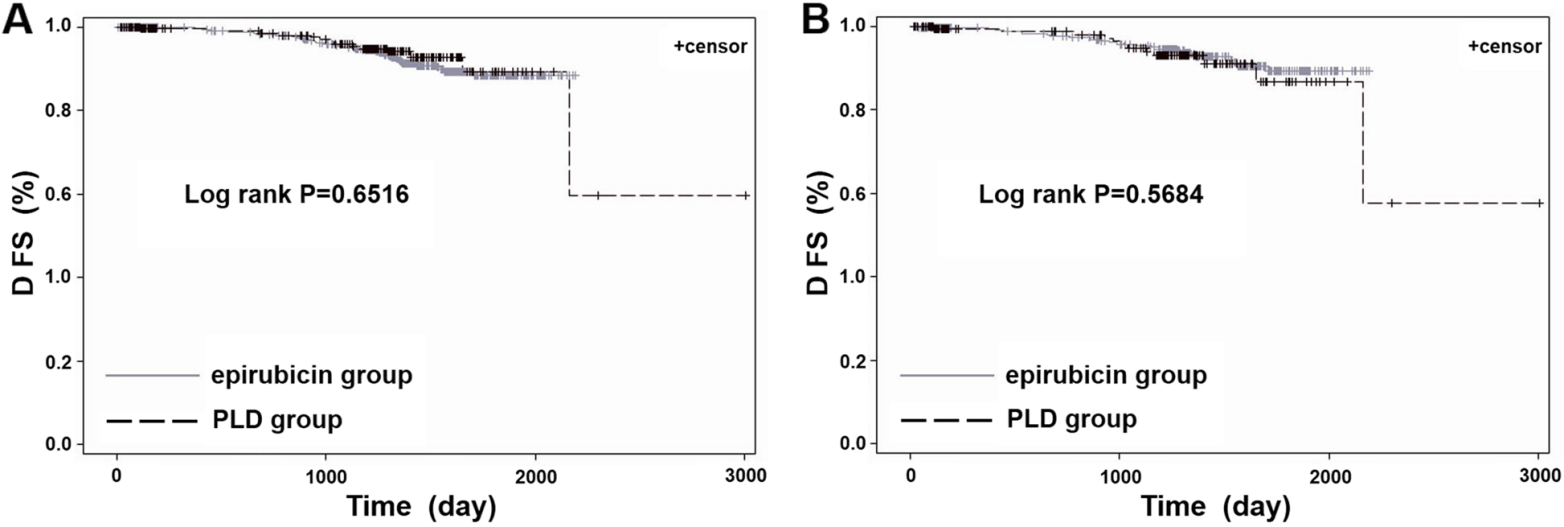
Kaplan-Meier curves of disease-free survival (DFS) of among patients who received adjuvant chemotherapy. (**A**) DFS before PSM, (**B**) DFS after PSM. PLD, pegylated liposomal doxorubicin

Before PSM, the 3-year DFS rate of the 939 eligible patients who received adjuvant chemotherapy was not significantly different between the PLD (96.0, 95% CI = 93.2–98.7%) and epirubicin groups (95.1, 95% CI = 93.1–97.1%) (*P* = 0.6516) (Fig. 2A and Table 5).

**Table 5:** Three-year DFS rate of adjuvant chemotherapy

	PLD group	Epirubicin group	*P* value	Rate difference (PLD group-epirubicin group), 95%CI
Before PSM, n	292	647		
3-year DFS, % [95%CI]	96.0% [93.2, 98.7]	95.1% [93.1, 97.1]	0.6516	0.85 [−2.54, 4.24]
After PSM, n	201	402		
3-year DFS, % [95%CI]	94.9% [91.1, 98.6]	95.4% [93.0, 97.9]	0.5684	−0.55 [−5.02, 3.92]

*PLD* Pegylated liposomal doxorubicin

After PSM, the 3-year DFS rate was also not significant different between the PLD (94.9, 95%CI = 91.1–98.6%) and epirubicin groups (95.4, 95% CI = 93.0–97.9%) (*P* = 0.5684) (Fig. 2B). The rate difference between the two groups and its 95% CI was − 0.55 [− 5.02, 3.92]. In the exploratory noninferiority analysis, the lower limit of the 95% CI was − 5.0% > − 10.0%, suggesting that the effectiveness of PLD is be not inferior to that of epirubicin (Table 5).

**Fig. 3:**
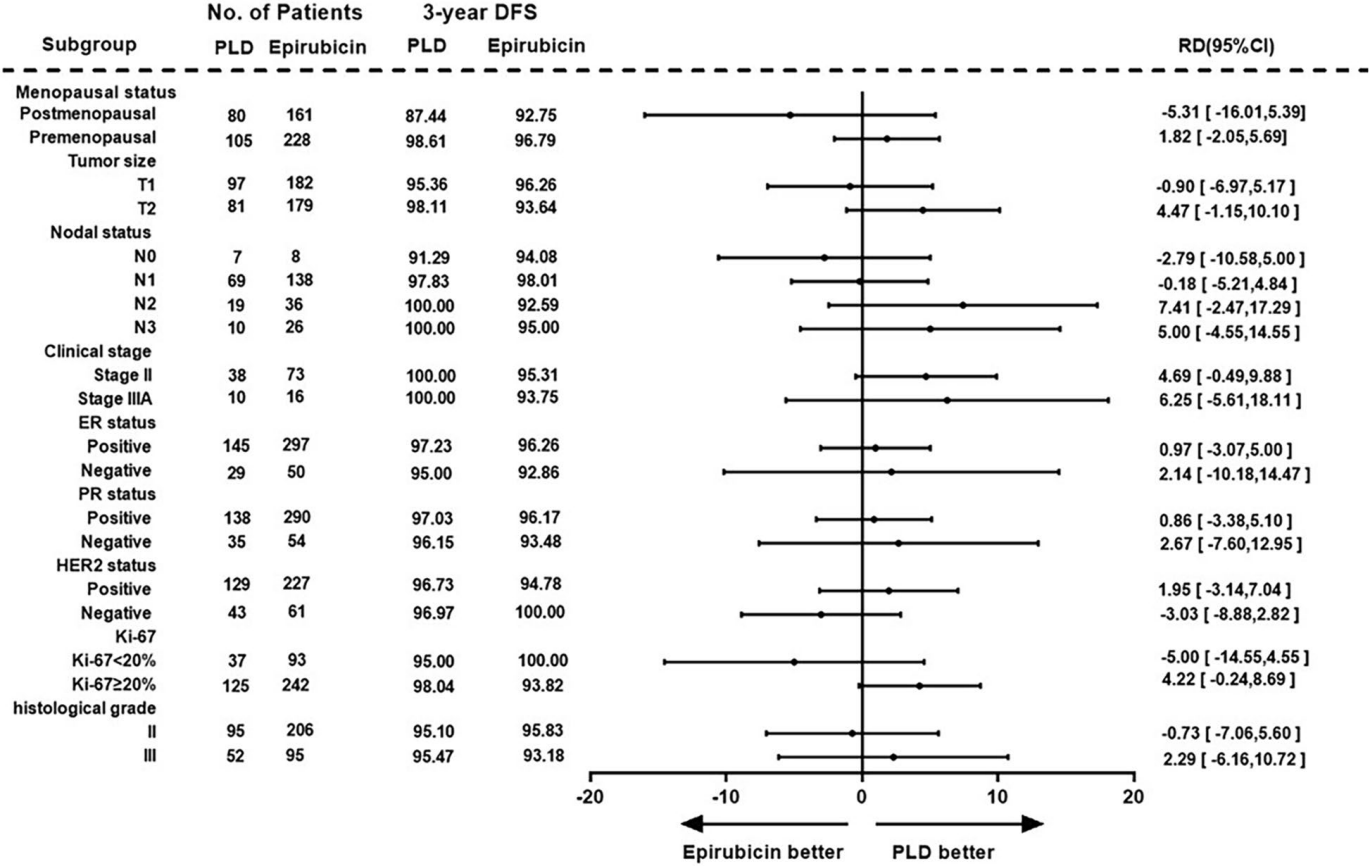
Forest plot of the 3-year DFS rate in key subgroups of adjuvant chemotherapy after PSM. RD, rate difference in 3-year DFS between the two groups

After PSM, the 3-year DFS rate was analyzed by subgroups according to the menopause status (premenopausal or postmenopausal), tumor size (T1 or T2), lymph node metastasis (N0, N1, N2 or N3), clinical stage (II or IIIA), ER (positive or negative), PR (positive or negative), HER-2 (positive or negative), Ki-67 expression (< 20% or ≥ 20%), and histological grade (grade II or III). 3-year DFS rate was higher in premenopausal, T2, N2, stage II, Ki-67 ≥ 20% patients in the PLD group than in the epirubicin group (Fig. 3).

#### Safety

A total of 1213 patients (362 in the PLD group and 851 in the epirubicin group) who received neoadjuvant chemotherapy or adjuvant chemotherapy were included in the safety analysis. According to the medical records of the patients, the incidence of adverse reactions was lower in the PLD group (15.2%) than in the epirubicin group (18.1%). The common adverse reactions were myelosuppression, decreased appetite, cardiotoxicity, and gastrointestinal reactions (Table 6). The incidence of cardiotoxicity was higher in the epirubicin group (6.6%) than in the PLD group (2.2%). The main manifestations of cardiotoxicity were abnormal ST segment (ECG), sinus tachycardia. There were no cardiac failure-related records. In addition, the incidences of myelosuppression, decreased appetite, alopecia, and gastrointestinal reaction were lower, but the incidence of nausea was higher in the PLD group than in the epirubicin group.

**Table 6:** Most common adverse reactions

n (%)	PLD group (*N* = 362)	Epirubicin group (*N* = 851)
Myelosuppression	19 (5.3%)	72 (8.5%)
Decreased appetite	8 (2.2%)	37(4.4%)
Cardiotoxicity	8 (2.2%)	56 (6.6%)
Gastrointestinal reaction	8 (2.2%)	31 (3.6%)
Alopecia	5 (1.4%)	29 (3.4%)
Nausea	21 (5.8%)	17 (2.0%)
D-dimer increase	0 (0.0%)	8 (0.9%)
Transaminase increase	0 (0.0%)	7 (0.8%)
Dizziness	1 (0.3%)	6 (0.7%)
Vomiting	0 (0.0%)	8 (0.9%)
Abnormal liver function	0 (0.0%)	4 (0.5%)

*PLD* Pegylated liposomal doxorubicin

## Discussion

Anthracycline plays an important role in the neoadjuvant and adjuvant treatment of breast cancer, and the common anthracycline-based chemotherapy regimens include AC, AC-T, TAC, and AT. Doxorubicin was the first anthracycline drug to be used in the treatment of breast cancer, and the common cardiotoxicity associated with doxorubicin is cardiac dysfunction [18]. Pegylated liposome doxorubicin (PLD) has unique pharmacokinetic and pharmacodynamic properties due to its altered formulation, which can effectively reduce drug exposure in normal tissue and thus minimize toxicity while ensuring treatment effectiveness [12].

pCR (ypT0/is or ypT0/is ypN0) is a standard effectiveness outcome of neoadjuvant therapy for breast cancer. Pooled analysis showed that patients who achieved pCR have improved survival [19, 20]. Previous studies have shown that the pCR of breast cancer patients after neoadjuvant chemotherapy is about 1–68% [21–23], varying according to the cancer subtype: 1% for luminal A, 8% for luminal B, 38% for HER2-positive, and 23% for triple-negative [23]. However, the clinical stage, HER2 status, Ki-67 expression, HR status, and other factors may affect the effectiveness of neoadjuvant therapy. Several studies have shown that PLD-containing neoadjuvant therapy is effective for the treatment of breast cancer [15, 24–28]. A retrospective study comparing the effectiveness and safety of PLD to epirubicin as neoadjuvant treatment for breast cancer demonstrated that patients in the PLD group had a similar clinical response rate (76.7% vs. 75.6%) and pCR rate (16.3% vs. 11.6%, *P* = 0.317) as those in the epirubicin group [29]. Yao et al. also found that PLD-containing neoadjuvant chemotherapy had comparable effectiveness (18.5% pCR rate) as epirubicin in the treatment of locally advanced breast cancer [22].

Adjuvant therapy is an important treatment for early breast cancer patients as it significantly reduces the risk of recurrence and improves patient survival [30–32]. Anthracycline-based chemotherapy is also a common adjuvant therapy [33, 34]. In the NEAT/BR9601 study, the seven-year follow-up results showed that compared with cyclophosphamide, methotrexate, and 5-fluorouracil (CMF) alone, CMF followed by epirubicin significantly improved the 5-year relapse-free survival (RFS) (78% vs. 71%, *P* < 0.0001) and 5-year OS rates (84% vs. 78%, *P* = 0.0007) of the 2391 breast cancer patients receiving adjuvant therapy [35]. In addition, several studies showed that PLD-based adjuvant treatment prolonged the DFS and improved the survival benefits of breast cancer patients. A study was conducted in stage I-III invasive breast cancer patients who received PLD adjuvant treatment, and the long-term follow-up results showed that the 5-year and 10-year DFS rates were 76.3 and 72.6%, respectively [36]. Another retrospective case-control study involving 103 patients with early breast cancer showed that effectiveness was similar between PLD- and epirubicin-based adjuvant treatment, and there was no significant difference in the 5-year DFS rate between the two groups [37]. Similar results were also observed in our study. The 3-year DFS rate of patients was comparable between the PLD and epirubicin groups (94.9% vs. 95.4%, *P* = 0.5684), and the lower limit of 95% CI of the rate difference between the two groups was − 5.0% > − 10.0%, which indicated that PLD was not inferior to epirubicin as adjuvant therapy for breast cancer. Nevertheless, the follow-up was only 3 years. The patients are still being followed, and the results will be updated.

There is increasing evidence that PLD can significantly reduce the risk of cardiotoxicity compared with other anthracyclines [12, 26, 38]. In our study, the incidence of cardiotoxicity was higher in the epirubicin group than in the PLD group (6.6% vs. 2.2%). In addition, compared with traditional doxorubicin, PLD resulted in lower incidences of nausea, vomiting, and myelosuppression [12]. The study by Yang et al. showed that the patients in the PLD group had lower incidences of grade 3 and 4 AEs than those in the epirubicin group, but hand-foot syndrome was more prevalent in the PLD group [26]. Consistent with previous findings, our results showed that the incidences of myelosuppression, decreased appetite, and gastrointestinal reaction were lower in the PLD group than in the epirubicin group. However, the hand-foot syndrome was not observed in the PLD group, which might be attributed to the integrity of the retrospective study data.

## Conclusions

In this study, we used a matched case-control design with stringent matching criteria to compare the effectiveness and safety of PLD vs. epirubicin as neoadjuvant or adjuvant chemotherapy in breast cancer patients who received the treatment within the same period. Patients who received adjuvant chemotherapy were followed up for at least 3 years to obtain their long-term survival benefit data. However, the long-term benefit of PLD is still unclear since the diagnosis and treatment data analyzed in this study were collected from only seven hospitals.

## Data Availability

The datasets used and/or analyzed during the present study are available from the corresponding author on reasonable request.

## Abbreviations

PLD: Pegylated liposomal doxorubicin
PSM: Propensity score matching
pCR: Pathological complete response
DFS: Disease-free survival
IARC: International Agency for Research on Cancer
CMF: Cyclophosphamide, methotrexate and 5-fluorouracil
RFS: Relapse-free survival
CI: Confidence interval
